# E14-F55 combination in M2 protein: a putative molecular determinant responsible for swine-origin influenza A virus transmission in humans

**DOI:** 10.1371/currents.RRN1044

**Published:** 2009-10-07

**Authors:** Chungen Pan, Shibo Jiang

**Affiliations:** ^*^Lindsley F. Kimball Research Institute, New York Blood Center and ^†^Lindsley F. Kimball Research Institute, New York Blood Center, New York, NY 10065, USA

## Abstract

The species-specific signatures of the swine-origin influenza A (H1N1) virus (S-OIV), which caused the current influenza pandemic, have not been well defined. By comparing the protein sequences of S-OIVs with those of swine, avian and human influenza viruses, we found that that almost all human IAVs and those causing influenza pandemics, including the 2009 S-OIVs, had a combination of glutamic acid (E) residue at position 14 and phenylalanine (F) residue at position 55 in their M2 protein, while only 22% and 4% of the swine and avian IAVs had the E14-F55 combination. These finding suggests that E14-F55 combination in the M2 protein of S-OIV may be a molecular determinant associated with its human-to-human transmission.

## Introduction

In mid-June of 2009, the World Health Organization declared the first influenza pandemic of the 21st century (www.who.int), which was caused by a novel swine-origin influenza A H1N1 virus (S-OIV) [1]. Several groups [1] [2] [3] [4] have extensively analyzed the gene sequences of the S-OIV strains, but they could not identify any molecular features previously shown to confer increased human-to-human transmissibility, suggesting that there might be some as yet unrecognized molecular determinants responsible for transmission of S-OIV in humans .

 By comparing the sequences of proteins of avian and human influenza A viruses (IAVs), Shih’s group previously identified a number of species-specific signatures of these viruses [5] . Most recently, Huang and colleagues examined the amino acid sequences of S-OIV A (H1N1) at these signature positions and found that most of the S-OIV protein sequences were avian-like signatures, although a few of them had changed from avian- to human-like signatures[6].

 Here we used a different approach to identify the potential species-specific signatures in S-OIV A (H1N1) by searching for both single and combined mutations, considering that some single mutations by themselves may not have a significant effect, but a combination of multiple mutations may have detrimental impact on viral transmissibility [7]. 

## Material and Methods

From the NCBI website (http://www.ncbi.nlm.nih.gov/genomes/FLU), we downloaded the sequences of ten proteins (HA, NA, PB1, PB2, PA, NP, NS1, NS2, M1 and M2) of human IAVs (3114 sequences), swine IAVs (139 sequences) and avian IAVs (1658 sequences), as well as those that caused the influenza A pandemics in 1918-1919 (H1N1)(2 sequences), 1957-1963 (H2N2)(23 sequences), 1968-1970 (H3N2)(57 sequences) and 2009 (H1N1)(803 sequences) [8]. We performed multiple alignments by using the ClustalX multiple sequence alignment program 1.83 [9]. 

## Results and Discussion 

We identified 159 amino acid residues in the ten proteins of human IAVs that were different from those at the corresponding positions in the proteins of avian and swine IAVs. Most of these residues in the proteins of 2009 S-OIVs are identical to those in avian and swine IAVs. Interestingly, however, two residues at positions 14 and 55 in the M2 protein of the 2009 S-OIVs are identical to human IAVs, *i.e.,* glutamic acid at position 14 (E14) and phenylalanine at position 55 (F55) , while most swine and avian IAVs contain glycine at position 14 (G14) and leucine at position (L55), respectively, although the entire M2 protein of S-OIVs had higher sequence identity with that of animal IAVs than human IAVs (**Figure 1**). By comparing the frequency of these two amino acid residues in all IAVs analyzed, we found that 100% of 2009 S-OIVs and >99% of human IAVs had E14 and F55 in the M2 protein. However, about 26% and 49% of the swine IAVs, and 63% and 29% of the avian IAVs had E14 and F55, respectively (Table 1). More interestingly, only 22% and 4% of the swine and avian IAVs had the E14-F55 combination, whereas 98.8% of human IAVs and 100% of the 2009 S-OIVs and those causing the 1957 and 1968 influenza pandemics had the E14-F55 combination  (Table 1). But the frequency (%) of the E14-F55 combination in M2 protein of the IAVs causing 1918 influenza pandemic cannot be calculated because there are only 2 related sequences available.

Our results suggest that the E14-F55 combination may be a molecular determinant responsible for human-to-human transmission of IAVs. It was reported that the M protein of S-OIV was derived from the Eurasian swine IAVs [3], in which the predominant residues at positions 14 and 55 are glycine (G14) and leucine (L55), respectively. Therefore, the double mutation of G14→E and L55→F may contribute to the increased human transmissibility of S-OIVs. 

M2 protein, which consists of an extracellular domain (M2e: aa 1-24), a transmembrane domain (TM: aa 25-43) and a cytoplasmic tail (CT: aa 44-97), plays important roles in the life cycle of IAV, as it forms disulfide-linked homotetrameric helix bundles to provide an acidified environment for virus uncoating and maturation by regulating the pH within the virion, thereby serving as a target for anti-influenza drugs [10]. It also regulates the pH of the trans-Golgi vesicles in IAV-infected cells to prevent the low pH-induced conformational change of the HA from occurring during its maturation [11] .  The fragment of residues 51–59 in the CT of M2 protein forms amphipathic helice [12] , an important part of the homotetrameric ion channel. Interestingly, the amino acid residue at the position of 55 is located in the middle of the amphipathic helice. The aromatic ring of F55 in ion channel may provide preferable environment for uncoating and maturation of human IAVs, while the aliphatic side chain of L55 in ion channel may give an environment suitable for the replication of swine IAVs (**Figure 2**). 

 M2e contains a conservative epitope that can induce antibodies capable of inhibiting virus budding and maturation and *in vivo* protection against both homologous and heterologous influenza viruses. Several M2e-based “universal” influenza vaccines are under preclinical and clinical development [5] [13] [14] . Liu et al have shown that the region of aa 10-20 in M2e protein confers the host restriction specificities [15] . It was reported that the amino acid residues at positions 16, 20, 28, 55, and 78 in M2 are associated with host specificity [16] . Since the functionality of M2 protein is determined by both M2e and CT sequences [12] , the combined mutation of residues in M2e, *e.g.,* E14, and CT, *e.g.,* F55, may have synergistic effect on the change of structure and function of the M2 protein as well as the shift of host restriction specificity, possibly rendering the virus more effective in infecting human cells. If this hypothesis is confirmed, E14, F55 and E14-F55 combination can serve as species-specific signatures for predicting cross-species transmission of the newly emerged IAVs and these sites may serve as targets for developing anti-IAV therapeutics and vaccines. 

Table 1. The frequency of E14, F55 and the E14-F55 combination in the M2 protein of human, swine, avian and pandemic IAVs

**Table:** 

Influenza A viruses	Frequency (%)		
	E14	F55	E14-F55
**S-OIV H1N1**	**721/721 (100)**	**721/721 (100)**	**721/721 (100)**
**Swine **	**36/139 (25.9)**	**66/135 (48.9)**	**29/135 (21.5)**
H1N1	12/60 (20.0)	22/60 (36.7)	10/60 (16.7)
H3N2	24/79 (30.4)	44/75 (58.7)	19/75 (35.3)
**Avian **	**1041/1658 (62.8)**	**471/1647 (28.6)**	**59/1647 (3.6)**
H1N1	1/42 (2.4)	2/41 (4.9)	1/41 (2.4)
H2N2	0/4 (0)	0/4 (0)	0/4 (0)
H3N2	1/53 (1.9)	14/53 (26.4)	0/53 (0)
H5N1	995/1064 (93.5)	28/1062 (2.6)	18/1062 (1.7)
H9N2	44/495 (8.9)	427/487 (87.8)	40/487 (8.2)
**Human**	**3081/3114 (98.9)**	**3105/3114 (99.7)**	**3076/3114 (98.8)**
H1N1	978/983 (99.5)	978/983 (99.5)	977/983 (99.4)
H3N2	2103/2131 (98.7)	2127/2131 (99.8)	2099/2131 (98.5)
**Past pandemic **	**81/81 (100)**	**80/82 (97.6)**	**80/82 (97.6)**
1968 H3N2	57/57 (100)	57/57 (100)	57/57 (100)
1957 H2N2	23/23 (100)	23/23 (100)	23/23 (100)
1918 H1N1	1/1*	0/2*	0/2*

*The frequency (%) cannot be calculated because there are only 2 related sequences available.

**Figure:**
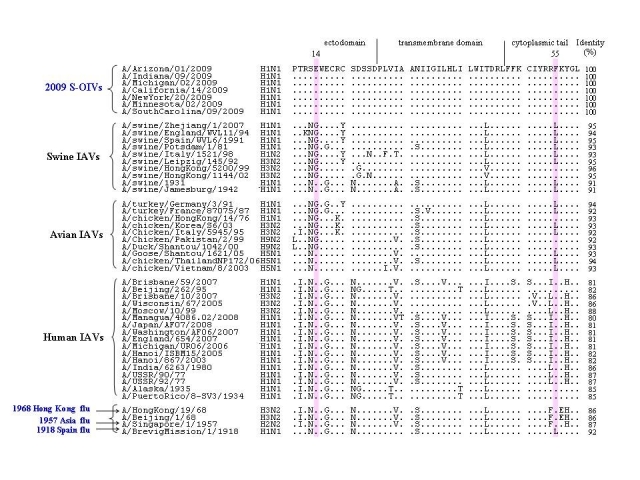

**Figure 1.** Amino acid sequence alignment of M2 proteins from the swine-original (H1N1) influenza virus (S-OIV) and other influenza A viruses.

**Figure:**
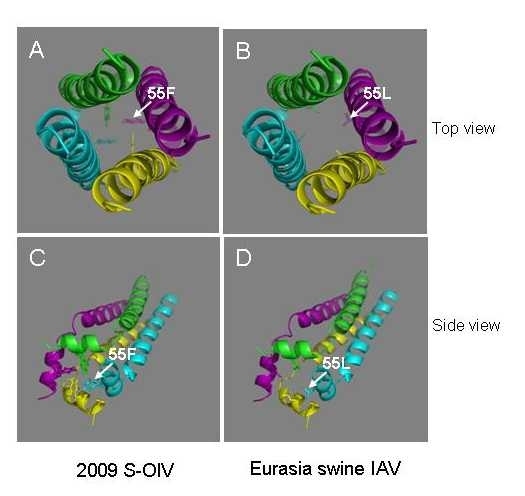

**Figure 2.** Top and side views of the homotetrameric helix bundles formed by CT of M2 protein from the 2009 S-OIV (A and C) and Eurasia swine IAV (B and D), respectively.

## Acknowledgments

The authors thank Mr. Byron Cheung for data collection.

## Funding information

C. Pan was partially supported by an Exchanging Scholarship from China Scholarship Council of the Ministry of Education of China.  

## Competing interests

The authors have declared that no competing interests exist.
